# Clinical and haematological predictors of antibiotic prescribing for acute cough in adults in Swiss practices – an observational study

**DOI:** 10.1186/s12875-015-0226-9

**Published:** 2015-02-06

**Authors:** Sven Streit, Peter Frey, Sarah Singer, Ueli Bollag, Damian N Meli

**Affiliations:** Institute of General Practice of the University of Bern, Gesellschaftsstrasse 49, 3012 Bern, Switzerland

**Keywords:** Acute cough, Antibiotics, Primary care of Switzerland, Point-of-care CRP testing

## Abstract

**Background:**

Acute cough is a common problem in general practice and is often caused by a self-limiting, viral infection. Nonetheless, antibiotics are often prescribed in this situation, which may lead to unnecessary side effects and, even worse, the development of antibiotic resistant microorganisms worldwide. This study assessed the role of point-of-care C-reactive protein (CRP) testing and other predictors of antibiotic prescription in patients who present with acute cough in general practice.

**Methods:**

Patient characteristics, symptoms, signs, and laboratory and X-ray findings from 348 patients presenting to 39 general practitioners with acute cough, as well as the GPs themselves, were recorded by fourth-year medical students during their three-week clerkships in general practice. Patient and clinician characteristics of those prescribed and not-prescribed antibiotics were compared using a mixed-effects model.

**Results:**

Of 315 patients included in the study, 22% were prescribed antibiotics. The two groups of patients, those prescribed antibiotics and those treated symptomatically, differed significantly in age, demand for antibiotics, days of cough, rhinitis, lung auscultation, haemoglobin level, white blood cell count, CRP level and the GP’s license to self-dispense antibiotics. After regression analysis, only the CRP level, the white blood cell count and the duration of the symptoms were statistically significant predictors of antibiotic prescription.

**Conclusions:**

The antibiotic prescription rate of 22% in adult patients with acute cough in the Swiss primary care setting is low compared to other countries. GPs appear to use point-of-care CRP testing in addition to the duration of clinical symptoms to help them decide whether or not to prescribe antibiotics.

## Background

Acute cough is one of the main reasons why patients see their General Practitioner (GP) [1]. Cough combined with other signs of respiratory infection, such as fever, rhinitis, sore throat, or discoloured sputum, is frequently caused by a virus, in which case treatment with antibiotics is not necessary. Antibiotics do not shorten the illness and may be associated with side effects [2,3]. Furthermore, the worldwide overuse of antibiotics is one of the main reasons for the development of resistance [4,5].

The antibiotic prescription rate in patients with acute cough varies largely in different countries, from 15% [6] to 83% [4]. Variations in antibiotic prescribing also exist in general practices within a country [7]. Different factors are known to increase the likelihood of prescription of antibiotics, such as the patient’s expectation [8,9], old age [10], female gender [11], and longer duration of symptoms [12,13]. The following GP-related factors also influence the prescription rate: the assumption that patients want antibiotics [14,15], more or less years in practice [15-17], and the license to self-dispense antibiotics [18]. Patients’ assumption that antibiotics help and the GPs’ fear of a devastating outcome are two more reasons why antibiotics are over-prescribed in patients with acute cough [8,9].

In a study conducted in Switzerland [19], 27% of patients with acute respiratory tract infection were treated with antibiotics, less compared to other countries. As in countries such as Scandinavian ones, the primary care system in Switzerland uses point of care devices in practices for full blood count (FBC) and C-reactive protein (CRP) testing, but about a third of all GPs have access to chest radiography in their own practice. Many practitioners are licensed to dispense medications, including antibiotics. Although many GPs act as gatekeepers to the health care system, patients are free to attend a physician of their choice, including specialists in secondary care. Accordingly there are no registered patient lists.

The aim of the present study was to assess factors associated with the prescription of antibiotics in patients presenting with acute cough and to scrutinize the role of point-of-care FBC, CRP testing and the immediate access to chest X-rays.

## Methods

### Study population

We focused on a sample of consecutive adult patients seeing their GPs because of a cough lasting less than 3 weeks. We excluded patients with asthma, chronic obstructive pulmonary disease (COPD), gastro-oesophageal reflux disease, the use of ACE-inhibitor, immunodeficiency or pregnancy. Medical students were recruited to collect the data during their clinical clerkships within general practice. Practices were located in and around the city of Bern, Switzerland.

### Processes and outcomes

Between September and November 2013, 39 medical students volunteered to record data on every adult patient attending a GP because of cough during their three-week GP attachments. Every student was only with one GP, forming a GP/student pair. Each pair of GP and student included a different number of patients (median 5, min. 1, max. 27). GPs were aware of the purpose of the study and the recording of data. After each consultation, the medical student and GP together assessed various baseline characteristics. These meetings allowed for the inclusion of data even if students occasionally were not present in every consultation, such as in the case of the students’ being occupied with other tasks, and also for teaching purposes, including the assessment of clinical signs, laboratory results and chest x-rays.

### Ethical considerations

This study did not interfere with the consultation between patients and GPs. Data available from the consultation were recorded anonymously. According to the cantonal ethical committee, no full ethical approval was needed for this study.

### Statistical analysis

The mean values and standard deviations of baseline characteristics were calculated. The Chi-square-test was used to compare categorical data and the Wilcoxon rank-sum (Mann–Whitney) test for non-parametric values. We used a mixed effects logistic regression model to derive odd ratios of prescription of antibiotics and adjusted for patient’s age, number of days of cough, haemoglobin level, WBC (white blood cell) count, and CRP measurements. GP’s age and ability to self-dispense antibiotics were also adjusted for. We selected these co-variables, because they were the most statistically significant differences between patients treated with antibiotics and without as seen in Table 1. Mixed effects logistic regression was used to account for multiple assessments within patient groups, and for clustering of patients depending on treating GPs. Lastly, using STATA release 13.1 (Stata Corp, College Station, TX, USA), we calculated a predictive model with the margins command to estimate the adjusted prediction of all statistically significant co-variables for the prescription of antibiotics. A p-value of 0.05 was considered to be statistically significant.

**Table 1:** **Patient characteristics**

**Characteristics** ^**a**^	**Overall** ^**b**^ **(n = 315)**	**Antibiotic treatment (n = 69)**	**No antibiotic treatment (n = 241)**	**p-value**
**Patients**				
Age, years (SD)	51.1 (18.2)	56.2 (19.0)	49.7 (17.8)	0.02
Women, n (%)	180 (57.1)	37 (53.6)	141 (58.0)	0.47
Smoker, n (%), n = 309	85 (27.5)	19 (27.9)	65 (27.3)	0.92
Nationality, n (%), n = 314				0.79
Switzerland	258 (82.2)	57 (82.6)	198 (82.5)	
Europe	43 (13.7)	10 (14.9)	31 (12.9)	
Other	13 (4.1)	2 (2.9)	11 (4.6)	
Demand for antibiotics^c^, n (%), n = 141	11 (3.4)	11 (15.9)	0 (0)	<0.001
**Symptoms**				
Days of cough, n (SD), n = 311	6.7 (4.5)	7.9 (4.9)	6.4 (4.4)	0.02
Sputum, n (%),	167 (53.0)	42 (60.9)	124 (51.5)	0.17
Rhinitis, n (%)	169 (53.7)	30 (43.5)	138 (57.3)	0.04
Sore throat, n (%)	103 (32.7)	18 (26.1)	84 (34.9)	0.17
Fever, n (%)	115 (36.5)	31 (44.9)	84 (34.9)	0.13
Comorbidities, n (%)				0.09
Cardiovascular	57 (18.1)	18 (26.1)	39 (16.2)	
Metabolic	12 (3.8)	4 (5.8)	7 (2.9)	
Other	33 (10.5)	9 (13.0)	24 (10.0)	
None	213 (67.6)	38 (55.1)	171 (71.0)	
**Findings**				
Lung auscultation, n (%), n = 314				0.001
Rhonchi	36 (11.5)	16 (23.2)	19 (7.9)	
Wheezing	29 (9.2)	10 (14.5)	19 (7.9)	
Other	29 (9.2)	6 (8.7)	22 (9.2)	
Normal auscultation	220 (70.1)	36 (53.7)	181 (74.8)	
Laboratory done, n (%)	219 (69.5)	56 (81.1)	162 (67.2)	0.03
Haemoglobin, g/l (SD)	141.0 (14.1)	136.3 (13.0)	143.0 (13.9)	0.003
White blood cells, G/l (SD)	7.3 (2.6)	8.7 (3.0)	6.9 (2.3)	<0.001
CRP, mg/dl (SD)	25.0 (38.8)	61.2 (60.5)	12.5 (12.3)	<0.001
Chest X-ray done, n (%)	36 (11.6)	22 (21.9)	14 (5.8)	<0.001
Infiltrate visible, n (%)	12 (33.3)	12 (54.6)	0 (0)	0.001

^a^For each characteristic with missing data, a label of “n” is provided behind each characteristic (e.g. Women, n).

^b^Five patients had missing data concerning antibiotic prescription.

^c^Patient opposed not to be prescribed antibiotics during consultation.

## Results

### Baseline characteristics

**Figure 1:**
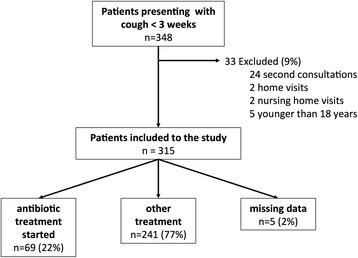
**Flowchart.**

Medical students registered 348 (3.2%) cases with acute cough out of 10’886 patients that they saw during their attachments. We excluded 33 patients (9%) for various reasons (Flowchart Figure 1). A fifth of patients (22%) were prescribed antibiotics; 78% were treated symptomatically.

Table 1 depicts the baseline characteristics of patients with acute cough. The two groups (with and without antibiotics) differed significantly with regard to age of patient, demand for antibiotics, days of cough, rhinitis, lung auscultation, haemoglobin level, WBC count, CRP measurement, and GPs’ self-dispensation of antibiotics. Table 2 shows characteristics of GPs and their offices.

**Table 2:** **Characteristics of GPs and their offices**

**General Practitioners** ^**a**^ **(n = 39)**	
Age, years, n (SD), n = 37	56.4 (6.8)
Experience^b^, n, years (SD), n = 38	20.7 (8.2)
Women, n (%), n = 37	6 (16.0)
Self-dispensing^c^, n (%), n = 37	30 (81.1)
Office location, n (%), n = 37	
City	11 (29.7)
Suburbs	5 (13.5)
Rural	21 (56.8)

^a^For each characteristic with missing data, a label of “n” is provided behind each characteristic (e.g. Women, n).

^b^Number of years working as a general practitioner.

^c^Ability to deliver and sell drugs directly at the GP office.

### Prediction of prescription of antibiotics

**Figure 2:**
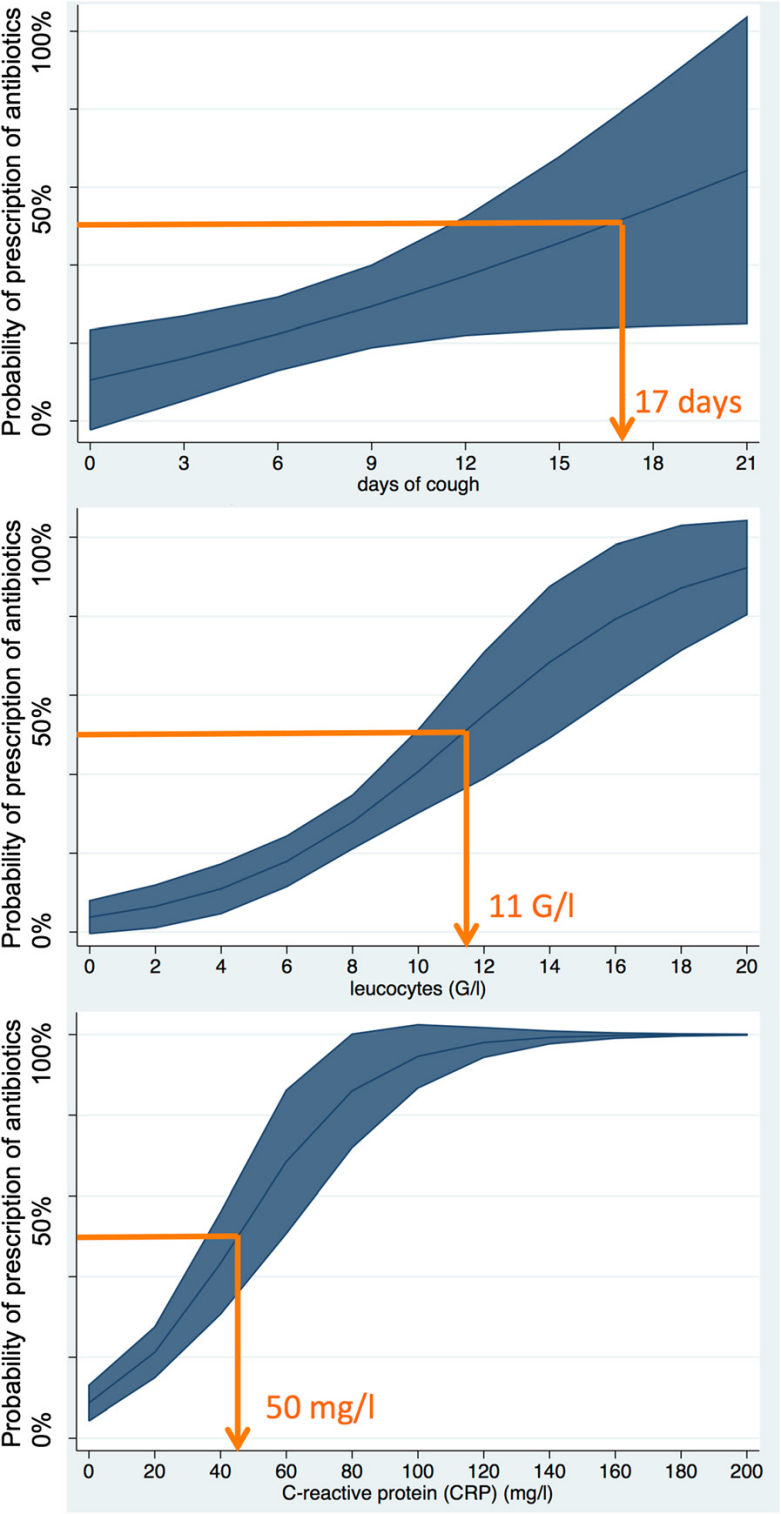
**Predictive models for threshold of prescription of antibiotics depending on days of cough, WBC count and CRP level.** *Example to read: following the horizontal line crossing the mean value (blue line) crossing x-axis e.g. 17 days (blue area =95% CI) as a predictive threshold of 17 days of cough for GPs to decide prescribing antibiotics.

After logistic regression, only the duration of cough (OR 1.13, 95% CI 1.02-1.27), WBC count (OR 1.33, 95% CI 1.06-1.68) and CRP (OR 1.07, 95% CI 1.03-1.10) emerged as significant predictors for the prescription of antibiotics. Figure 2 shows a predictive model to estimate adjusted prediction for the prescription of antibiotics. Threshold mean values for the prescription of antibiotics are 17 days of cough, leukocyte count of 11 G/l and CRP value of 50 mg/l.

## Discussion

In this study, 39 medical students collected data from over 300 patients with acute cough during their three-week clerkships in general practice. A fifth of all patients were prescribed antibiotics. The baseline characteristics of the patients who were or were not prescribed antibiotics differed significantly with regard to age of the patient, the demand of the patient for antibiotics, the duration of cough, rhinitis, lung auscultation, haemoglobin content, white blood cell count, CRP measurement, dispensing of antibiotics by the GPs themselves, and the medical student’ presence during consultation. After regression analysis, however, only three variables remained as firm predictors for the prescription of antibiotics – the duration of cough, WBC count and CRP level.

Blood testing was not performed on all the patients, perhaps due to the effect of the severity of illness on physicians’ decisions to use laboratory tests. Patients who were prescribed antibiotics and had CRP levels checked had a significantly higher CRP than those who were treated symptomatically. Point of care testing for CRP has been shown to be a useful adjunct to clinical decision making in primary care [19]. CRP tests are widely available in Swiss GP practices. They have proven to be of great assistance in deciding whether antibiotics for acute respiratory infections are indicated or not [20]. The usefulness of CRP testing in acute cough and respiratory tract infection for reducing antibiotic prescription has been assessed repeatedly. There is general agreement about the influence of point-of-care CRP results on whether or not to prescribe antibiotics in these situations [21-23]. The views about the diagnostic value of CRP in infections of the lower respiratory tract are, however, controversial [24-26]. André *et al.* questioned the use of CRP in patients with respiratory tract infections [27]. For patients assigned viral diagnoses, antibiotic prescription increases with increasing duration of symptoms and increasing value of CRP, but in 59% of the patients assigned viral diagnoses, CRP values were not interpreted correctly resulting in a non-optimal use of antibiotics.

A raised leukocyte count has been found to be a non-specific, unreliable indicator of respiratory infection [28]. In this study, however, WBC counts proved to be a reliable predictor for the prescription of antibiotics. This may be due to the fact that automated lab-machines often measure CRP and WBC levels together even though GPs focus on CRP. In our study, 61% of all patients had both laboratory tests. However, CRP was measured in 69% of the cases and WBC counts in 62%. Similarly, GPs are tempted to prescribe antibiotics with the increasing duration of cough although it has been shown that the median duration of untreated cough is up to 18 days [29]. The mismatch between patients’ expectations and the reality of the natural history of acute cough may have important implications for antibiotic prescription. Moreover there is little evidence that antibiotics change the number of days with cough in patients with non-complicated acute bronchitis and discoloured sputum [30-32].

Some Swiss practices have a license to dispense pharmaceuticals, including antibiotics, thus linking diagnostic and therapeutic procedures. This is convenient for patients as they do not need to pay separate visits to pharmacies. The reverse side is that practitioners with dispensaries have financial incentives to prescribe medication. Nevertheless, the availability of laboratory, X-ray, and other diagnostic and therapeutic facilities within practices may be partially responsible for the fact that Switzerland is one of the leaders regarding the restricted use of antibiotics and the country’s high ranking on the Euro Health Consumer Index, with regard to the interval between diagnosis and treatment for a great number of health care aspects [33]. For example, looking at chest X-rays on Table 1, it can be seen that GPs’ feeling the need for such X-rays in order to make a therapeutic decision (antibiotics yes/no) is significantly linked with the prescription of antibiotics, especially when infiltrate visible. The same applies to abnormal sounds from lung auscultation. It can be surmised that acute cough in these patients was seen as the expression of lower respiratory tract infection, e.g. pneumonia.

This cross-sectional study conducted by motivated students and GPs has produced findings which can be considered representative of the national picture, as the equipment of GP practices in Switzerland is quite uniform with regard to the availability of point-of-care laboratory tests, including CRP and blood count, X-ray, lung function tests and electrocardiogram monitors. It enables GPs to reach an accurate diagnosis within a few minutes and to tailor therapeutic medications, especially antibiotics. This is important in view of the ever increasing resistance to antibiotics in Europe [34].

### Strengths and weaknesses

This is the first study about predictors of antibiotic prescribing in adult primary care patients with medical students collecting clinical and haematological data during clerkships. Consultations conducted in the presence of students may be considered unrepresentative as GPs might be concerned to set a good example. This is rather unlikely as students were present during the consultation in 86% of all cases but there was no correlation between student presence and GPs’ prescription of antibiotics (p = 0.96). However, the number of patients included was relatively small, and the data quality suffered from some missing data in the data entry process. This may be due to the fact that laboratory investigations were not undertaken in more than around 60% of all patients. The severity of illness may have influenced this decision. Lastly, we did not check on the adequacy of GPs’ prescription of antibiotics over and above the impact of clinical findings and biomarkers.

## Conclusions

Medical students collected data from adult patients presenting with acute cough during their three-week clerkships with GPs. Antibiotics were found not to be prescribed as often as in some other countries. CRP level, WBC count and duration of cough turned out to be the main predictors of antibiotic prescription suggesting that GPs use the results of these tests in making treatment decisions.

## Abbreviations

CRP: C reactive protein
FBC: Full blood count
GP: General practitioner
WBC: White blood cells

## References

[1] Westert G, Jabaaij L, Schellevis F (2006). Morbidity, Performance and Quality In Primary Care.

[2] Fahey T, Stocks N, Thomas T (1998). Quantitative systematic review of randomised controlled trials comparing antibiotic with placebo for acute cough in adults. BMJ.

[3] Butler CC, Hood K, Verheij T, Little P, Melbye H, Nuttall J (2009). Variation in antibiotic prescribing and its impact on recovery in patients with acute cough in primary care: prospective study in 13 countries. BMJ.

[4] Smucny J, Fahey T, Becker L, Glazier R. Antibiotics for acute bronchitis. Cochrane Database Syst Rev*.* 2004; 4:CD000245 10.1002/14651858.CD000245.pub2 15494994

[5] Arroll B, Kenealy T. Antibiotics for the common cold and acute purulent rhinitis. Cochrane Database Syst Rev*.* 2005; 3:CD000247. 10.1002/14651858.CD000247.pub2 16034850

[6] Pan Y, Henderson J, Britt H (2006). Antibiotic prescribing in Australian general practice: how has it changed from 1990–91 to 2002-03?. Respir Med.

[7] Ashworth M, Charlton J, Ballard K, Latinovic R, Gulliford M (2005). Variations in antibiotic prescribing and consultation rates for acute respiratory infection in UK general practices 1995–2000. Br J Gen Pract.

[8] Chung A, Perera R, Brueggemann AB, Elamin AE, Harnden A, Mayon-White R (2007). Effect of antibiotic prescribing on antibiotic resistance in individual children in primary care: prospective cohort study. BMJ.

[9] Macfarlane J, Holmes W, Macfarlane R, Britten N (1997). Influence of patients’ expectations on antibiotic management of acute lower respiratory tract illness in general practice: questionnaire study. BMJ.

[10] Kroening-Roche JC, Soroudi A, Castillo EM, Vilke GM (2012). Antibiotic and bronchodilator prescribing for acute bronchitis in the emergency department. J Emerg Med.

[11] Gonzales R, Steiner JF, Sande MA (1997). Antibiotic prescribing for adults with colds, upper respiratory tract infections, and bronchitis by ambulatory care physicians. Jama.

[12] Andre M, Odenholt I, Schwan A, Axelsson I, Eriksson M, Hoffman M (2002). Upper respiratory tract infections in general practice: diagnosis, antibiotic prescribing, duration of symptoms and use of diagnostic tests. Scand J Infect Dis.

[13] Dosh SA, Hickner JM, Mainous AG, Ebell MH (2000). Predictors of antibiotic prescribing for nonspecific upper respiratory infections, acute bronchitis, and acute sinusitis. An UPRNet study. Upper Peninsula Research Network. J Fam Pract.

[14] van der Horst HE, Berger MY (2012). [Patients expect antibiotics. Or not? A folie a deux]. Ned Tijdschr Geneeskd.

[15] Akkerman AE, Kuyvenhoven MM, van der Wouden JC, Verheij TJ (2005). Determinants of antibiotic overprescribing in respiratory tract infections in general practice. J Antimicrob Chemother.

[16] Lam TP, Lam KF (2001). Management of upper respiratory tract infection by family doctors. Int J Clin Pract.

[17] Cadieux G, Tamblyn R, Dauphinee D, Libman M (2007). Predictors of inappropriate antibiotic prescribing among primary care physicians. Cmaj.

[18] Huang N, Chou YJ, Chang HJ, Ho M, Morlock L (2005). Antibiotic prescribing by ambulatory care physicians for adults with nasopharyngitis, URIs, and acute bronchitis in Taiwan: a multi-level modeling approach. Fam Pract.

[19] Hersberger KE, Botomino A, Sarkar R, Tschudi P, Bucher HC, Briel M (2009). Prescribed medications and pharmacy interventions for acute respiratory tract infections in Swiss primary care. J Clin Pharm Ther.

[20] Briel M, Young J, Tschudi P, Hersberger KE, Hugenschmidt C, Langewitz W (2006). Prevalence and influence of diagnostic tests for acute respiratory tract infections in primary care. Swiss Med Wkly.

[21] Cals JW, Schot MJ, de Jong SA, Dinant GJ, Hopstaken RM (2010). Point-of-care C-reactive protein testing and antibiotic prescribing for respiratory tract infections: a randomized controlled trial. Ann Fam Med.

[22] Jakobsen KA, Melbye H, Kelly MJ, Ceynowa C, Mölstad S, Hood K (2010). Influence of CRP testing and clinical findings on antibiotic prescribing in adults presenting with acute cough in primary care. Scand J Prim Health Care.

[23] Andreeva E, Melbye H (2014). Usefulness of C-reactive protein testing in acute cough/ respiratory tract infection. An open cluster-randomized clinical trial with C-reactive protein testing in the intervention group. BMC Fam Pract.

[24] Van der Meer V, Neven AK, van den Broek PJ, Assendelft WJ (2005). Diagnostic value of C reactive protein in infections of the lower respiratory tract: systematic review. BMJ.

[25] Engel MF, Paling FP, Hoepelman AI, van der Meer V, Oosterheert JJ (2012). Evaluating the evidence for the implementation of C-reactive protein measurement in adult patients with suspected lower respiratory tract infection in primary care: a systematic review. Fam Pract.

[26] Van Vugt SF, Broekhuizen BD, Lammens C, Zuithoff NP, de Jong PA, Coenen S (2013). Use of serum C reactive protein and procalcitonin concentrations in addition to symptoms and signs to predict pneumonia in patients presenting to primary care with acute cough; diagnostic study. BMJ.

[27] André M, Schwan A, Odenholt I, Swedish Study Group on Antibiotic Use (2004). The use of CRP tests in patients with respiratory tract infections in primary care in Sweden can be questioned. Scand J Infect Dis.

[28] Chalmers JD, Hill AT (2011). Investigation of “non-responding” presumed lower respiratory tract infection in primary care. BMJ.

[29] Ebell MH, Lundgren J, Youngpairoj S (2013). How long does a cough last? Comparing patients’expectations with data from a systematic review of the literature. Ann Fam Med.

[30] Moore M, Stuart B, Coenen S, Butler CC, Goossens H, Verheij TJ (2014). Amoxicillin for acute lower respiratory tract infection in primary care: subgroup analysis of potential high-risk groups. Br J Gen Pract.

[31] Llor C, Moragas A, Bayona C, Morros R, Pera H, Plana-Ripoli O (2013). Efficacy of anti-inflammatory or antibiotic treatment in patients with non-complicated acute bronchitis and discoloured sputum: randomised placebo controlled trial. BMJ.

[32] Butler CC, Kelly MJ, Hood K, Schaberg T, Melbye H, Serra-Prat M (2011). Antibiotic prescribing for discoloured sputum in acute cough/ lower respiratory tract infection. Eur Respir J.

[33] Euro Health Consumer Index (EHCI) 2013. [http://www.healthpowerhouse.com]

[34] Goossens H, Ferech M, Vander Stichele R, Elseviers M (2005). Outpatient antibiotic use in Europe and association with resistance: a cross-national database study. Lancet.

